# A Computational Procedure for Atomistic Modelling of Polyphosphazenes towards Better Capturing Molecular-Level Structuring and Thermo-Mechanical Properties

**DOI:** 10.3390/polym14071451

**Published:** 2022-04-02

**Authors:** Kay Chen, Baris Demir

**Affiliations:** 1 Institute for Nanoscale Science and Technology, Flinders University, Adelaide, SA 5042, Australia; 2 Centre for Defence Chemistry, Cranfield University, Defence Academy of United Kingdom, Shrivenham SN6 8LA, UK; 3 Centre for Theoretical and Computational Molecular Science, The Australian Institute for Bioengineering and Nanotechnology, The University of Queensland, Brisbane, QLD 4072, Australia

**Keywords:** Poly(phosphazenes), chain-growth polymerisation, molecular dynamics simulations, thermo-mechanical properties

## Abstract

Poly(phosphazenes)(PZ) are versatile polymers due to their tunable properties that can be tailored for specific applications. Despite extensive experimental research, not all properties are tested, and the list of PZs studied via molecular simulations is limited. Further, a general procedure to generate and test PZ systems is lacking. We present an in situ polymerization procedure developed to make, test, and tune the thermo-mechanical properties of four PZs—poly(dichlorophosphazene)(PZ-DC), poly[bis(2,2,2-trifluoroethoxy)]phosphazene (PZ-TFE), poly(2,2,2-trifluoroethoxy-5,6-diazidohexanoxy) phosphazene (PZ-Azido), and poly(2,2,2-trifluoroethoxy-5,6-dinitratohexanoxy)phosphazene (PZ-Nitrato) via molecular dynamics simulations. The predicted thermo-mechanical properties (i.e., density and glass transition temperature) agreed with experimental values when a direct comparison of PZ systems was possible. This demonstrates the reproducibility and reliability of our procedure which will help understand the behaviour of PZs at the molecular scale.

## 1. Introduction

Polyphosphazenes (PZ) are inorganic-organic hybrid polymers that consist of an alternating phosphorous-nitrogen backbone (-P=N-) with two organic side groups attached to the phosphorous atom. PZs have been used in applications such as fuel cells [1,2], flame retardant materials [3,4,5], polymer bonded explosives [6], and drug delivery systems [7,8]. The extensive applications of PZs stem from the wide-ranging thermo-mechanical properties they possess i.e., high thermal stability and low glass transition temperature [9]. By functionalising the side chains with different moieties, one can tune the properties of the PZ, as the side chains play a significant role in determining the properties of the resulting polymer [10,11,12].

For example, in a recent work, Han et al. [13] synthesised PZs with tetraphenylphosphonium cation functionalised polymer chains to provide better alkaline stability for anion exchange membranes [2]. In a different application, Clubb et al. [14] investigated PZs as alternative polymer binders for environmentally friendly rocket propellants. Their work examined the influence of side chain lengths and functional groups (azido and nitrato) attached to the end of side chains on the thermal and decomposition properties.

Although hundreds of PZs have been synthesised, experimental data for complete thermo-mechanical properties of PZs are limited and at times contradictory [15]. Computer simulations such as molecular dynamics (MD) have become valuable tools to provide insight into molecular-level interactions to establish structure-property relationships. MD simulations of PZs have been used to predict properties such as glass transition temperature [16,17], solubility parameter [18], thermal degradation [19], and density [18]. A variety of force fields have been used to simulate PZs such as DRIEDING [20], AMBER [19], COMPASS [21], and CHARMM [22]. MD simulations can therefore be used to predict thermo-mechanical properties when experimental data is unavailable. Although numerous publications have studied PZs via MD simulations, the procedures used to generate polymer chains were rather primitive, and the development of computational procedures for modelling PZs is limited.

In a review, Fried [23] compiled studies where molecular simulations were used to investigate various properties of PZs. In none of the work reported in Fried’s review, a dynamic polymerisation procedure to make PZs and predict their properties (where the PZs were used as polymer medium) was reported. For instance, Sun et al. [21] parameterised and validated the COMPASS forcefield for PZs to predict the properties of three poly(dibutoxyphosphazenes) isomers. They found good agreement between the densities and glass transition temperatures obtained from MD simulations and experimental data. Their initial polymer structures were generated using a stepwise chain construction scheme based on the conventional RIS model using the Insight/Amorphous cell program. Similarly, Kroger and Fried [18] investigated properties (density, glass transition temperature, and solubility parameter) of four PZs of biomedical relevance. An atactic chain with 150 repeat units was built in Materials Studio for each PZ. After polymer construction, a periodic cell was created using the Amorphous Cell module within Materials Studio. A single chain of the polymer was used to construct each amorphous cell. In very recent work, Wang et al. [17] used MD simulations to predict the effect of side groups on the glass transition temperatures of poly(ethoxy/phenoxy)phosphazenes. In their procedure, nine polymer chains with different molar ratios of ethoxy/phenoxy groups based on probability construction and 150 repeat units (determined by the Fox-Flory equation) were constructed prior to MD simulations. This shows that bond formation between monomers was not captured on the fly, or in other words, the bonds between monomers were not formed during the simulations.

The procedures reported above to make and test polymer samples are not robust or easily followed due to a lack of detail. Additionally, the above procedures use one polymer chain to construct their simulation cell to predict macroscopic properties. This is an unreliable method as it does not consider the different end to end polymer interactions that influence properties in real experimental systems. Furthermore, those procedures are not versatile for testing the ultimate properties of PZs as a function of the degree of polymerisation (DP) as their repeat units are fixed. These examples show that a dynamic polymerisation procedure is needed to enhance our knowledge of PZs and tune them for different applications, by varying the functionality and architecture of PZs and DP.

Our work aims to bridge this gap. We have developed a robust computational procedure for making and testing PZs using MD simulations. This is the first MD work that focuses on modelling PZs starting from the simplest to custom-made monomers. The novelty of our work is the procedure itself where we start the polymerisation process at different points of the liquid monomer system to generate physically reliable polymer samples. Furthermore, our protocol can capture the distribution of polymer chains. Lastly, our procedure is flexible as it can be used with any force field.

**Figure 1:**
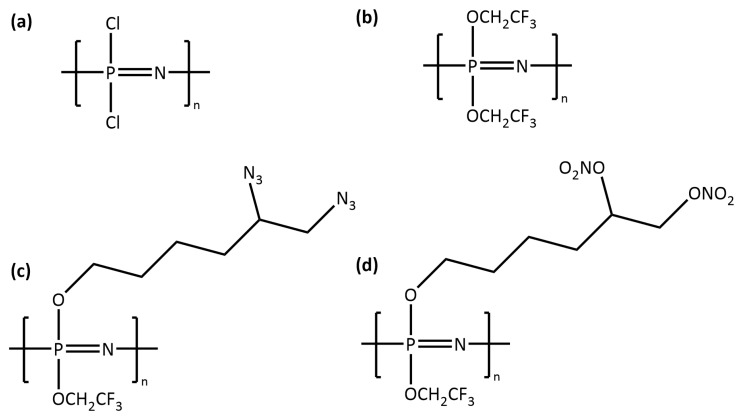
Molecular structures of repeat units: (**a**) PZ-DC, (**b**) PZ-TFE, (**c**) PZ-Azido and (**d**) PZ-Nitrato.

In this study, four PZs were chosen (Figure 1). The first was poly(dichlorophosphazene) (PZ-DC) which is a reactive intermediate for the synthesis of other PZ polymers [24]. The second was poly[bis(2,2,2-trifluoroethoxy)]phosphazene (PZ-TFE), which has been studied using MD simulations and has sufficient experimental characterisation available. The third and fourth PZs were poly(2,2,2-trifluoroethoxy-5,6-diazidohexanoxy)phosphazene (PZ-Azido) and poly(2,2,2-trifluoroethoxy-5,6-dinitratohexanoxy)phosphazene (PZ-Nitrato). PZ-Azido and PZ-Nitrato are energetic PZs that have been studied for propellant applications that lack experimental data for mechanical properties [14]. It should be highlighted that no MD simulation work on the polymerisation and characterisation of the PZ-Azido and PZ-Nitrato has been reported in the literature. Our work contributes characterisation for unavailable thermo-mechanical properties and expands the list of PZs that have been investigated via MD simulations.

Our procedure is based on a five-step modelling procedure that enables a systematic approach to build polymer structures in situ via chain-growth polymerisation and test their thermo-mechanical properties. First, the four repeat units (Figure 1) were built, geometry optimised, and used for the calculation of partial atomic charges via the charge equilibration method [25,26]. Second, a simulation cell was generated for each system, and a simulated annealing (SA) procedure was applied to adequately equilibrate the systems [27]. Third, a chain growth polymerisation procedure [28] was applied to generate polymer samples. Fourth, thermal properties (i.e., glass transition temperature (T${}_{g}$) and coefficients of volumetric thermal expansion (CVTEs)) were predicted via an isobaric-isothermal cooling procedure. Finally, the mechanical properties (i.e., Young’s modulus and Poisson’s ratio) were predicted by deforming the polymerised samples under uni-axial tensile deformation. A future perspective has also been provided regarding how to extend the procedure reported herein to future applications of PZs.

## 2. Models and Methodology

### 2.1. Simulation Details

Molecular dynamics (MD) simulations were carried out using the DREIDING force field with Lennard-Jones potential [29]. We used a generic force field that uses generalised parameters and force constants as different force fields can influence simulation results because of their parameters. The Nosé-Hoover thermostat [30,31] and barostat [30,32] were used to control the simulation temperature and pressure, respectively. A cut-off distance of 12 Å was considered for the calculations of the long-range van der Waals (vdW) and Coulombic interactions. A tail correction and the particle-particle-particle-mesh (PPPM) algorithm [33] were used for the calculations of Coulombic interactions between charged atoms. A time-step of 1 fs was used for the integration of the Newton’s equations of motion (EoM). Periodic boundary conditions (PBCs) were considered in all directions. LAMMPS simulation software package (version: 16Mar18) (lammps.sandia.gov, accessed on 23 February 2022) [34] and Visual Molecular Dynamics (VMD) [35] were utilised to perform MD simulations and visualise simulation samples, respectively.

### 2.2. Preparation of Liquid Samples

The monomers, PZ-DC, PZ-TFE, PZ-Azido and PZ-Nitrato (Figure 1) were generated using AVOGADRO software [36], and geometry optimised until the difference between two successive iterations for energy calculations was less than 1 × 10${}^{-10}$ kcal·mol${}^{-1}$. The geometry optimised initial structures were used in the partial atomic charge (PAC) calculations. PACs were calculated prior to running the simulations as the DREIDING force-field does not include PACs per se. A charge equilibration method (QEq) that was reported in the literature and tested on various systems [26,37] was used [25]. Briefly, a geometry optimised initial structure (i.e., a monomer) was placed in a cubic simulation cell with a dimension of 200 Å, and the PACs were calculated. All PACs are reported in Figure S1 and Tables S1–S4. Upon the completion of partial charge calculations, a liquid sample for each system was generated. 2000 of each type of monomer were randomly placed in a cubic simulation cell with a dimension of 200 Å using PACKMOL software [38].

The low density liquid samples were used in the MD simulations. A geometry optimisation using the FIRE algorithm [39], performed over 10,000 steps to overcome the potential overlaps of atoms that could happen during the random packing of monomers. Later, the temperature of each sample was increased to 50 ${}^{\circ }$C for 50 ps and kept at this temperature for 100 ps using the NVT-MD ensemble. Next, a 500 ps simulation was run in the NPT-MD ensemble to equilibrate the density of each sample. Following this, a simulated annealing (SA) procedure [26,40] was used to ensure adequate mixing of monomers and adoption of energetically favoured configurations. Each sample was then cooled from 227 to 50 ${}^{\circ }$C. The temperature was kept at 50 ${}^{\circ }$C for 0.2 ns, followed by a temperature ramp to 227 ${}^{\circ }$C over a period of 0.2 ns. Each sample was kept at 227 ${}^{\circ }$C for 1 ns. The samples were then cooled to 50 ${}^{\circ }$C over a time period of 0.5 ns. A simulation in the NPT-MD ensemble was performed at 50 ${}^{\circ }$C for 0.2 ns to ensure that after the SA cycle the sample density was adjusted accordingly. In the last step of the SA cycle, a simulation of 0.2 ns in the NVT-MD ensemble was carried out to record a trajectory of 1 frame for every 1000 steps for analysis (i.e., calculation of the radial distribution functions (RDFs)).

### 2.3. Polymerisation Step

Once the liquid samples were adequately equilibrated via the SA procedure, a homopolymerisation protocol reported in the literature was initiated [41]. In this protocol, 2% of monomers were randomly selected as initiators through which polymers chains were grown [27,28]. Once 1% of available monomers reacted, the polymerising sample was relaxed via a multi-step relaxation procedure. The length of new bonds was gradually decreased while the bond force constants were gradually increased from a deliberately low value to the value defined in the force field in 20 steps using the NVT-MD ensemble. Table S5 provides the bond lengths and bond force constants used in each step. Once 95 % of available monomers were reacted, the polymerisation protocol was terminated. The amorphous polymerised samples were named with a prefix of ‘p-’ (i.e., p-PZ-DC, p-PZ-Azido).

### 2.4. Analysis and Prediction of Thermo-Mechanical Properties

Upon the completion of the polymerisation protocol, the polymerised samples were cooled with a cooling rate of 20 ${}^{\circ }$C·ns${}^{-1}$ from 80 to −90 ${}^{\circ }$C to predict thermal properties. At each temperature point (10 ${}^{\circ }$C) a 0.5 ns simulation was performed in the NPT-MD ensemble, and an averaged density value was recorded for every 1 ps simulation (amounting to 500 density values at each temperature value). T${}_{g}$ is determined by the intersection of two lines that are fitted using a piecewise function to the density versus temperature plot [42]. Coefficients of volumetric thermal expansion (CVTE) for regions above and below T${}_{g}$ were calculated using the slope of each fitted line to the reduced volume versus temperature plot.

Once cooled to 27 ${}^{\circ }$C, the polymerised samples were deformed under a uni-axial tensile deformation with a strain rate of 5 × 10${}^{-7}$ s${}^{-1}$ at 27 ${}^{\circ }$C and 1 atm, while keeping the cell dimensions in the other two directions free to adjust. One simulation was carried out for each direction (in total three simulations per system). The deformation simulations were used to generate a stress-strain curve (SSC) for each system. Both the tensile stress and tensile strain were averaged over every 1000 steps. The linear region of each SSC (2.0% of strain) was used to calculate Young’s modulus and Poisson’s ratio.

## 3. Results and Discussion

We acknowledge here that certain thermo-mechanical properties have not been experimentally reported for the PZ systems studied in this paper. Further, of those experimentally available, some differed in morphology for example, than the PZs in this work. Therefore, our predicted results may not be directly comparable to those experimentally reported. Comparisons with experimental values will be discussed where possible.

### 3.1. Liquid Samples

**Figure 2:**
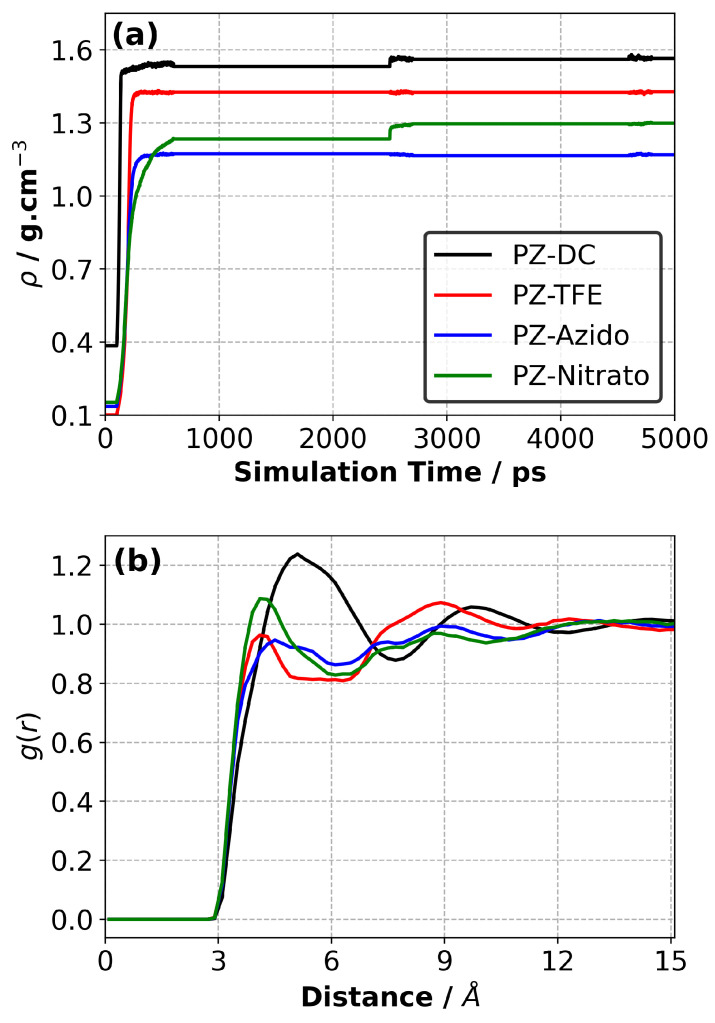
(**a**) Density evolution of liquid samples at 50 ${}^{\circ }$C and 1 atm. (**b**) Radial distribution function between head (P) and tail (N) of PZ monomers.

Figure 2a shows the density evolution for the liquid samples during the simulated annealing (SA) procedure. There are two regions where the density was readjusted due to the reorganisation of molecules facilitated by the SA cycles run at elevated temperatures. Two cycles of SA were found to be sufficient to obtain equilibrated samples because the change in the density during the second SA cycle (during the NPT part) was minor. Therefore, the SA procedure was stopped after two cycles.

The density evolution plots indicate the PZ-DC system had the highest density of 1.565 g·cm${}^{-3}$, followed by PZ-TFE, PZ-Nitrato, and PZ-Azido (Table 1). This can be attributed to the fact that the PZ-DC monomer is the smallest among the four monomers investigated and can pack better compared to the other monomers. In addition, the PZ-DC monomer contains two chlorine atoms which contribute to the high density of the liquid PZ-DC system. Further, it is shown that as the length of carbon side chains increases, the density of the system decreases. Longer side chains introduce greater rotational freedom which limits the packing of monomers, leading to lower density [43]. This trend was observed when one of the -CF${}_{3}$CH${}_{2}$O groups in the PZ-TFE monomer was replaced by a longer carbon chain as seen in PZ-Azido and PZ-Nitrato, irrespective of their pendant functional groups. Although PZ-Azido and PZ-Nitrato possessed the same monomer structures with different functional groups, PZ-Nitrato exhibited a higher density than PZ-Azido. This can be due to the increased polarity of the -ONO${}_{2}$ groups compared to the -N${}_{3}$ groups which increase the intermolecular interactions and therefore packing between monomers, resulting in a higher liquid density [44].

The RDF plots obtained between the head and tail atoms (i.e., P and N atoms, respectively) for the equilibrated liquid systems at 50 ${}^{\circ }$C are shown in Figure 2b and indicate that the first peak ends around 6 Å (except the PZ-DC system) whereas a larger distance (around 8 Å) was observed for the PZ-DC system. The results show that the amplitude of the first peak for the PZ-TFE and PZ-Azido was slightly smaller than unity, whereas it is slightly larger for the PZ-Nitrato system, suggesting a similar distribution of P-N atoms of the monomers. On the other hand, the location of the first peak for the PZ-DC system was larger (∼1.2 Å) than the other three systems. This suggests the average distance between the reactive head and tail atoms were more distant from each other in the PZ-DC system compared to the other three systems.

To ensure all samples were generated in a similar way during the polymerisation process, a reaction cut-off distance for the homopolymerisation was chosen to be 6 Å. However, when the rate in the number of new bonds decreased substantially, the reaction cut-off distance was increased gradually by 0.5 Å until a degree of polymerisation of 95% was achieved for each system.

**Figure 3:**
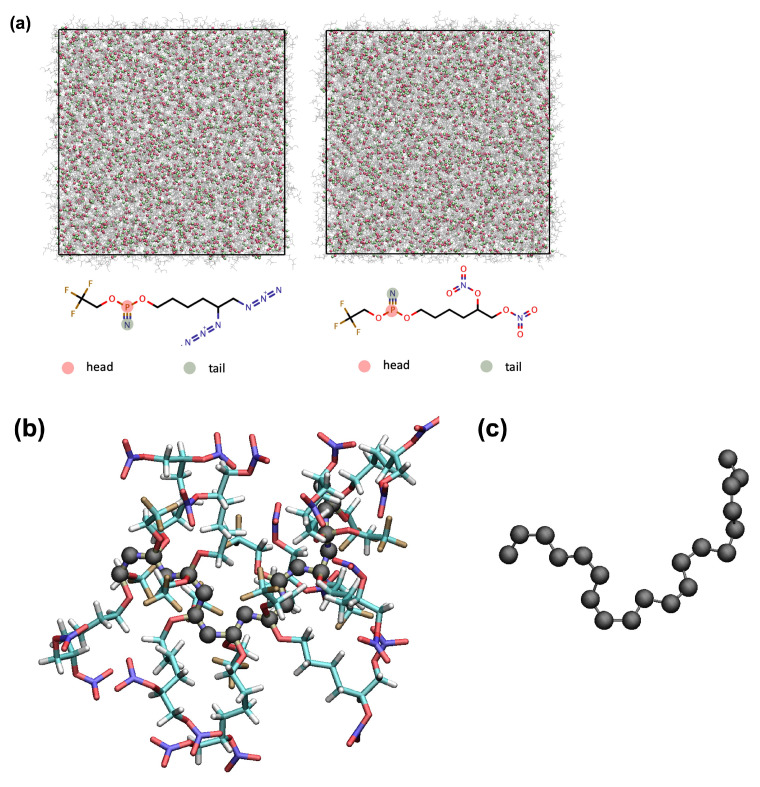
(**a**) A snapshot for the liquid PZ-Azido and PZ-Nitrato systems taken at the completion of the simulated annealing procedure at 50 ${}^{\circ }$C. (**b**) A polymer chain (up to 10 mers) of p-PZ-Nitrato. (**c**) The backbone of the polymer chain shown in (**b**).

A snapshot taken at the completion of the SA procedure for the PZ-Azido and PZ-Nitrato systems is reported in Figure 3a. The head and tail atoms were shown as red and green spheres, respectively, demonstrating that the reactive sites were distributed equally in the simulation cell. Therefore, once the polymerisation procedure starts, polymer chains begin developing through the reactive monomers (chosen randomly) found in the simulation cell. Upon the completion of the SA procedure, a protocol was initiated to start polymerisation. Figure 3b shows a snapshot for a part of the polymer chain taken from the polymerising p-PZ-Nitrato system. The visual inspection indicates that the two alike side chains were organised in a *trans* form. Figure 3c shows the backbone of the polymer chain (i.e., the covalently connected P and N atoms), shown in Figure 3b.

### 3.2. Thermal Properties

**Figure 4:**
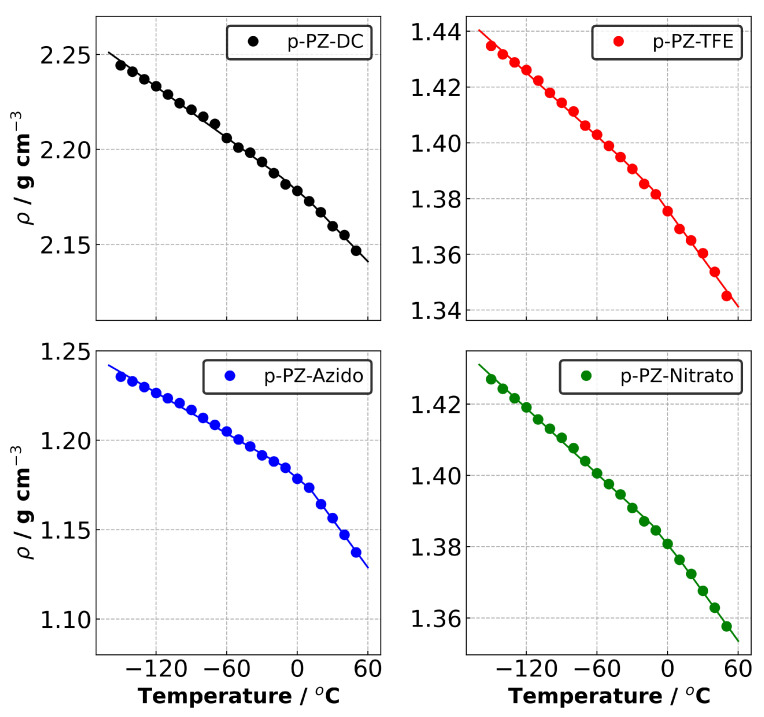
Density versus temperature data for each polymerised system.

The polymerised samples are named with the prefix ‘p-’. For example, the polymer system based on PZ-Azido monomers is named p-PZ-Azido. Figure 4 shows the density versus temperature plots obtained during the isobaric-isothermal cooling process. A similar trend was observed between the experimental and our polymerised and liquid densities of PZ-Nitrato and PZ-Azido, where PZ-Nitrato displayed higher densities than PZ-Azido in every case.

The thermo-mechanical properties are summarised in Table 2. The predicted densities of p-PZ-DC and p-PZ-Nitrato obtained a reasonable agreement with the experimental densities of 1.98 and 1.53 g·cm${}^{-3}$ at 25 ${}^{\circ }$C, respectively [6,45]. In the case of p-PZ-TFE, there was no reported experimental data for amorphous PZ-PTFE; however, a density of 1.707 g·cm${}^{-3}$ at 25 ${}^{\circ }$C was reported for 60% crystalline p-PZ-PTFE [46]. Based on available experimental data, Fried et al. [16] estimated the amorphous phase density of p-PZ-TFE to be 1.65 g·cm${}^{-3}$ and using MD simulations, predicted 1.633 g·cm${}^{-3}$. Our density of 1.365 g·cm${}^{-3}$ was lower than Fried et al.’s estimated amorphous density. The difference between our result and Fried et al.’s predicted density can be attributed to different polymer construction methods and force fields. However, we highlight that the estimated amorphous density by Fried et al. is contradicted by Nagai et al. [47]. In the work done by Nagai et al., they measured an experimental density of 1.82 g·cm${}^{-3}$ for ∼34% crystalline p-PZ-TFE [47]. They argue that since p-PZ-TFE is similar to poly(4-methyl-1-pentene), lower density in crystalline regions than those in amorphous regions would be present. This was observed in their results as they had lower crystallinity (∼34%) compared to Hirose et al. [46]. (60%) which gave a higher density.

For p-PZ-Azido, our density was below the experimental value of 1.43 g·cm${}^{-3}$. One factor in the lack of agreement is due to the different ratio of diazidohexanoxy side group present. As our monomer structure was fixed, our generated polymers contained a 50/50 proportion of trifluroroethoxy and diazidohexanoxy side groups, whereas the experiment reports 44% diazidohexanoxy as the highest. We estimate that at 50% diazidohexanoxy, a lower experimental density would result. This is based on decreasing densities from 1.52 g·cm${}^{-3}$ at 20% to 1.43 g·cm${}^{-3}$ at 44% reported by Golding et al. [6]. Furthermore, increased diazidohexanoxy side groups would lower the density due to the presence of a longer carbon chain from the diazidohexanoxy which affects the packing of polymer chains. In that case, our predicted density may have improved experimental agreement as the systems would have the same side group ratio. We note here that experimental density values can vary due to factors such as sample preparation, instrument choice, and accuracy [48].

We also examine the percentage of change in density between the highest and lowest temperatures. This evaluates the material integrity which is important for composite applications where operating temperatures can vary substantially. The results show that the highest change in density was observed for p-PZ-TFE and p-PZ-Azido, where p-PZ-Nitrato indicated the lowest change in density. Comparison of the two systems with energetic groups shows that the p-PZ-Nitrato has both a higher density and lower change in density compared to p-PZ-Azido, indicating greater material integrity with respect to temperature changes.

**Figure 5:**
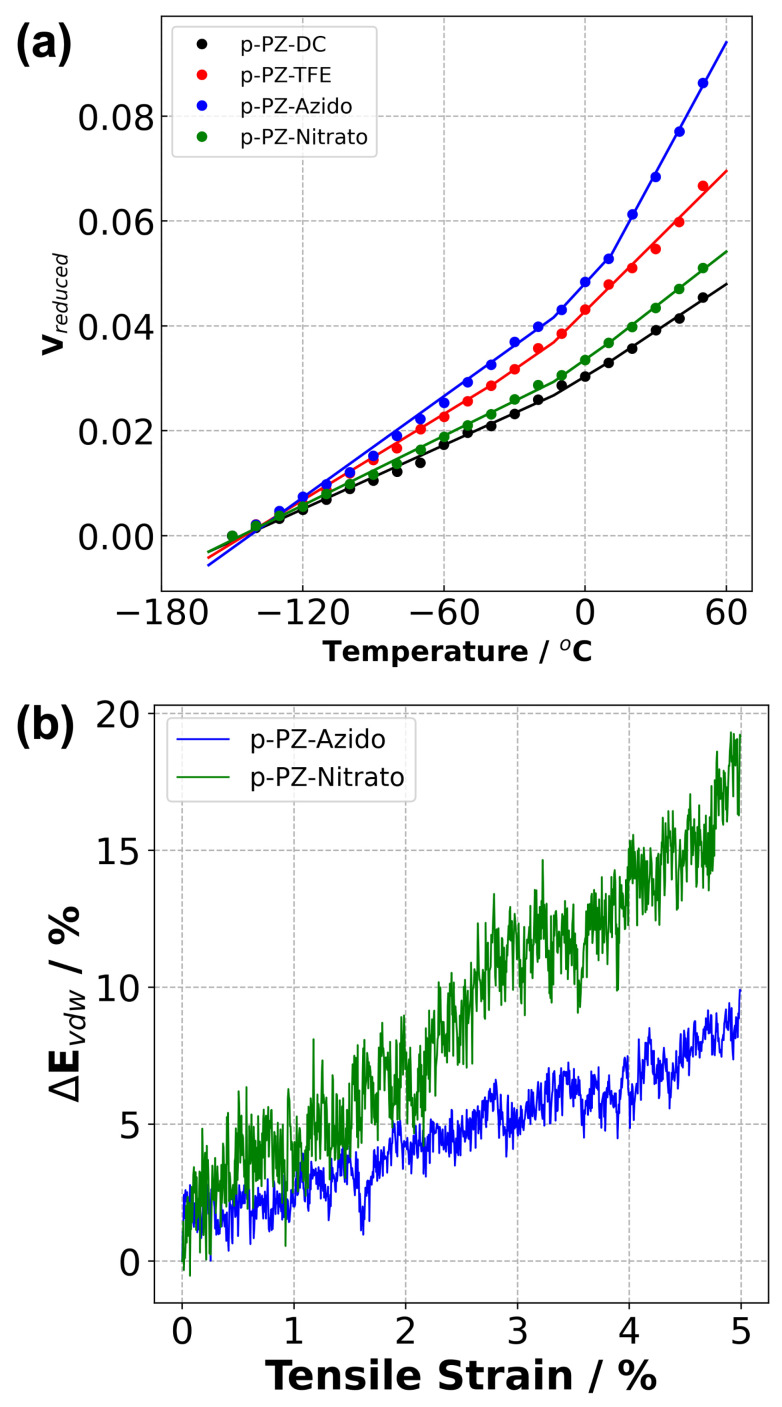
(**a**) Reduced volume versus temperature data for each polymerised system. (**b**) Change in vdW energy for the p-PZ-Azido and p-PZ-Nitrato systems in response to applied constant strain at 20 ${}^{\circ }$C.

T${}_{g}$ and CVTE of each system were calculated using the density versus temperature (Figure 4) and reduced density versus temperature plots (Figure 5), respectively. A correction term is applied to adjust predicted T${}_{g}$ values due to the discrepancy between the cooling rate implemented in our simulations (20 ${}^{\circ }$C·ns${}^{-1}$) and those set in the experimental measurements. The correction term subtracts 3 ${}^{\circ }$C from the predicted T${}_{g}$ values per order of magnitude difference in cooling rates [49]. The corrected T${}_{g}$ values are reported in Table 2 and named T${}_{g,corr.}$.

Our predicted values are in reasonable agreement with the experimental values of p-PZ-Nitrato at −43 ${}^{\circ }$C, and p-PZ-TFE which had a reported T${}_{g}$ range of −53 to −82 ${}^{\circ }$C [46,50,51,52]. A predicted T${}_{g}$ of −61.2 ${}^{\circ }$C for p-PZ-TFE was reported by Fried et al. [16] and showed greater agreement with the range of reported T${}_{g}$ than our predicted result. This is possibly due to their use of the COMPASS force field which had been previously parametrised and validated for PZ systems [21]. Despite a greater agreement in T${}_{g}$, their procedure of making polymer samples is limited, as a polymer sample composed of one single polymer chain is not representative enough to compare with real-life polymer systems. For example, T${}_{g}$ can be affected by the interactions between polymer chain ends which would not be accounted for in Fried et al. [43].

There was significantly less agreement with experimental T${}_{g}$ of −59 ${}^{\circ }$C [6] for p-PZ-Azido and p-PZ-DC which had a reported T${}_{g}$ range of −58 to −66 ${}^{\circ }$C [45,51,52]. This can be attributed to the lack of comparable experimental data as discussed above. Further, variations in experimental T${}_{g}$ can be attributed to different measurement methods, cooling rates, monomer side chain compositions, and molecular weight of polymers [43]. This is seen with p-PZ-TFE where torsional braid analysis measured a T${}_{g}$ of −53 ${}^{\circ }$C [52], which was closer to our predicted value.

We also observed the following trends: (1) our predicted T${}_{g}$ results were higher than the experimental T${}_{g}$ for all PZ systems; (2) the T${}_{g}$ values followed a non-monotonic behaviour for both experimental and predicted values with response to changes in side chain length.

CVTEs were examined to gain insight into the behaviour of polymeric materials and the dimensional stability of a structure at elevated temperatures. CVTEs were predicted using the reduced volume versus temperature data (Figure 5a) and reported in Table 2. The comparison of the four systems revealed that p-PZ-Azido showed the highest CVTE for both above and below T${}_{g}$ in the amorphous and glassy regions, respectively. This indicates greater polymer chain expansion facilitated by the azido groups in response to temperature change compared to p-PZ-Nitrato.

### 3.3. Mechanical Properties

Each polymerised sample was deformed under uni-axial tensile deformation, where an external constant strain was applied on. The applied strain caused the internal stress to increase in the polymerised systems. Figure S2 shows the tensile stress versus tensile strain plot for each system obtained at 20 ${}^{\circ }$C. Our computational mechanical test results indicate that larger stress developed in the p-PZ-DC and p-PZ-Nitrato samples than p-PZ-TFE and p-PZ-Azido during the uni-axial tensile deformation. There was a significant difference between the predicted Young’s modulus for p-PZ-TFE of 1.09 GPa, and those experimentally reported ranging from 0.110 to 0.216 GPa [53,54]. This may be due to the discrepancy between the simulated and experimental strain rates. Therefore, we focus on the observed trends rather than the absolute values of Young’s modulus for the PZ systems.

The results indicate that modifying the parent monomer (PZ-TFE) with a diazidohexanoxy side group (PZ-Azido) increased the polymer’s tensile response by ∼50%, and ∼228% with dinitratohexanoxy (PZ-Nitrato). It was observed that Young’s modulus for the p-PZ-Nitrato was more than double of p-PZ-Azido. The greater polarity of the -ONO${}_{2}$ group may account for this as p-PZ-Nitrato and p-PZ-Azido only differ in the functional group attached to the hexanoxyl side chain ends (Figure 1) [55]. As Young’s modulus is also a function of intermolecular forces, an increased polarity would result in stronger intermolecular interactions between polymer chains, resulting in a stiffer material [56]. This is supported by the evolution of energy change during the uni-axial tensile deformations. The results indicate that the largest difference between the energy response of these two systems under uni-axial deformation was observed in the van der Waals (vdW) interactions, as reported in Figure 5b. The analysis showed that the change in vdW energy was larger for the p-PZ-Nitrato than the p-PZ-Azido. This means that the p-PZ-Nitrato system requires more energy for vdW interactions to break. Therefore, this imparts a higher Young’s modulus to the p-PZ-Nitrato system.

In addition to Young’s modulus, Poisson’s ratio is calculated using the tensile strain-strain curves and reported in Table 2. Poisson’s ratio is a parameter indicating the tendency of a material to expand in directions orthogonal to the direction of strain applied on the material. Our simulation results show that Poisson’s ratio of p-PZ-Azido (0.47) was the largest followed by p-PZ-TFE (0.41). Poisson’s ratio for p-PZ-Nitrato (0.33) was similar to p-PZ-DC (0.31).

Considering the results in Table 2 and those experimentally reported, we observe that p-PZ-Azido exhibits greater flexible elastomeric properties than p-PZ-TFE through the diazidohexanoxy group. This is indicated by its low T${}_{g}$ and Young’s modulus, and higher CVTE and Poisson’s ratio. However, applications of p-PZ-Azido may be limited due to its high CVTE and change in density. In contrast, the presence of the dinitratohexanoxy group in p-PZ-Nitrato results in a stiffer elastomer which is reflected by its higher Young’s modulus and T${}_{g}$, and lower Poisson’s ratio and CVTE.

## 4. Conclusions and Future Directions

This paper reports a detailed procedure to make and test PZs using MD simulations. Our computational procedure is reproducible and versatile as it provides sufficient details to allow the reproduction of our results at each step in the process and can be used with any forcefield. The physical and thermo-mechanical properties of four PZs (PZ-DC, PZ-TFE, PZ-Azido, and PZ-Nitrato) were predicted. Additionally, this is the first report of energetic PZs (PZ-Azido and PZ-Nitrato) using MD simulations.

Our protocol proved reliable when our simulated systems were 1:1 with the experimental systems. This is evident in the p-PZ-Nitrato system where we obtained reasonable agreement (∼±10% of the experimental value) as the experimental system had a ratio of 51% dinitratohexanethoxy side groups and our MD simulations had 50%. Additionally, using a generic force field with our procedure, we were able to achieve experimental agreement similar to those who have used parameterised and validated forcefields for PZ systems. This shows that our protocol can be used to generate and model PZ systems that are more experimentally representative to better capture the molecular-level behaviour of these polymers.

**Figure 6:**
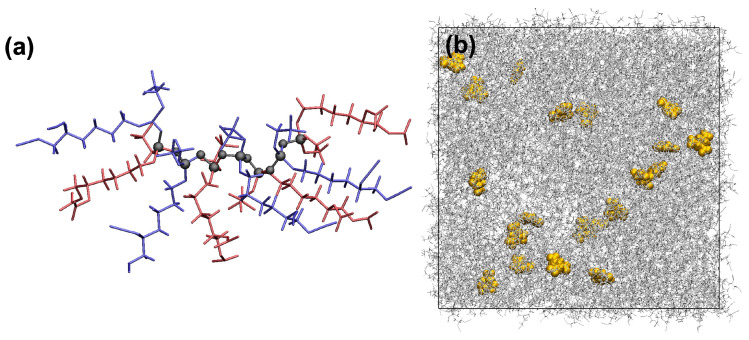
(**a** A representative polymer chain comprised of alternated monomers of PZ-Azido (in blue) and PZ-Nitrato (in red). The black spheres represent the reacted head and tail atoms. (**b**) A representative simulation cell where RDX molecules (in yellow) were randomly distributed in the p-PZ-Azido system (in grey).

The computational procedure developed and reported herein will provide a basis for a series of future research directions, that include: (i) polymer chains grown in the presence of multiple types of monomers [57]. Figure 6a shows a schematic of a polymer chain comprised of an alternated combination of PZ-Azido and PZ-Nitrato monomers; (ii) mixed polymer systems for composite applications. Figure 6b shows a representative simulation cell of randomly distributed RDX molecules in the p-PZ-Azido system; (iii) the influence of the degree of polymerisation in the PZs and cross-linked PZs with hardeners with various architecture and functionality (i.e., aliphatic and aromatic hardeners).

## Figures and Tables

**Table 1:** Densities of liquid systems at 50 ${}^{\circ }$C in g·cm${}^{-3}$.

System	ρ
PZ-DC	1.565
PZ-TFE	1.428
PZ-Azido	1.169
PZ-Nitrato	1.298

**Table 2:** Densities and thermo-mechanical properties of polymerised samples. Densities at 20 ${}^{\circ }$C in g·cm${}^{-3}$, T${}_{g,corr.}$ in ${}^{\circ }$C and CVTEs in ×10${}^{-4}$ K${}^{-1}$, Young’s modulus in GPa, Poisson’s Ratio (in absolute numbers) unitless.

	p-PZ-DC	p-PZ-TFE	p-PZ-Azido	p-PZ-Nitrato
Density	2.167	1.365	1.164	1.372
Change in Density	0.123	0.171	0.169	0.100
T${}_{g,corr.}$	−32.6	−51.8	−29.8	−39.5
CVTE (<T${}_{g,corr.}$)	2.03	2.74	3.22	2.21
CVTE (>T${}_{g,corr.}$)	2.99	4.46	8.29	3.49
Young’s Modulus	4.19	1.09	1.56	3.58
Poisson’s Ratio	0.31	0.41	0.47	0.33

## Supplementary Materials

The following supporting information can be downloaded at: https://www.mdpi.com/article/10.3390/polym14071451/s1. Figure S1. Partial atomic charges used for (a) PZ-DC, (b) PZ-TFE, (c) PZ-Nitrato, (d) PZ-Azido. Figure S2. (a) Tensile stress-strain curves (SSCs) for the polymerised systems obtained at 20 oC. (b) A zoomed-in version of (a). The lines represent the fit lines used to calculate the Young’s modulus, up to a strain of 2%. Table S1. List of the partial atomic charges for the unique atomic sites for the PZ-DC (the labels corresponding to Figure S1). Table S2. List of the partial atomic charges for the unique atomic sites for the PZ-TFE (the labels corresponding to Figure S1). Table S3. List of the partial atomic charges for the unique atomic sites for the PZ-Azido (the labels corresponding to Figure S1). Table S4. List of the partial atomic charges for the unique atomic sites for the PZ-Nitrato (the labels corresponding to Figure S1). Table S5. Bond length and bond force constants used for relaxing newly formed bonds between the monomers for each system.

## Abbreviations

The following abbreviations are used in this manuscript:

PZ	Polyphosphazenes
MD	Molecular dynamics
PAC	Partial atomic charge
SA	Simulated annealing
RDF	Radial distribution function
CVTE	Coefficient of volumetric thermal expansion
T${}_{g}$	Glass transition temperature
