# Supplementary material for: A Computational Procedure for Atomistic Modelling of Polyphosphazenes towards Better Capturing Molecular-Level Structuring and Thermo-Mechanical Properties
Source: Polymers (Basel). 2022 Apr 2;14(7):1451. doi: 10.3390/polym14071451 (PMC9002744; doi:10.3390/polym14071451)

Supporting Information

A Computational Procedure for Atomistic Modelling of Polyphosphazenes towards Better Capturing Molecular-level Structuring and Thermo-mechanical Properties

Kay Chen[a] and Baris Demir[b,c]

[a] Flinders Institute for Nanoscale Science and Technology, Flinders University, Bedford Park, SA, Australia

E-mail: kay.chen@flinders.edu.au

[b] Australian Institute for Bioengineering and Nanotechnology (AIBN), The University of Queensland, Brisbane, Queensland 4072, Australia

[c] Centre for Defence Chemistry, Cranfield University, Defence Academy of United Kingdom, Shrivenham, SN6 8LA, UK

E-mail: b.demir@uq.edu.au

Contents

**Partial atomic charges**	**pS2-pS3**
**Bond length-bond force constants used during the equilibration**	**pS4**
**Tensile stress-strain curves for the polymerised systems**	**pS5**

Partial atomic charges

Figure S1. Partial atomic charges used for (a) PZ-DC, (b) PZ-TFE, (c) PZ-Nitrato, (d) PZ-Azido.

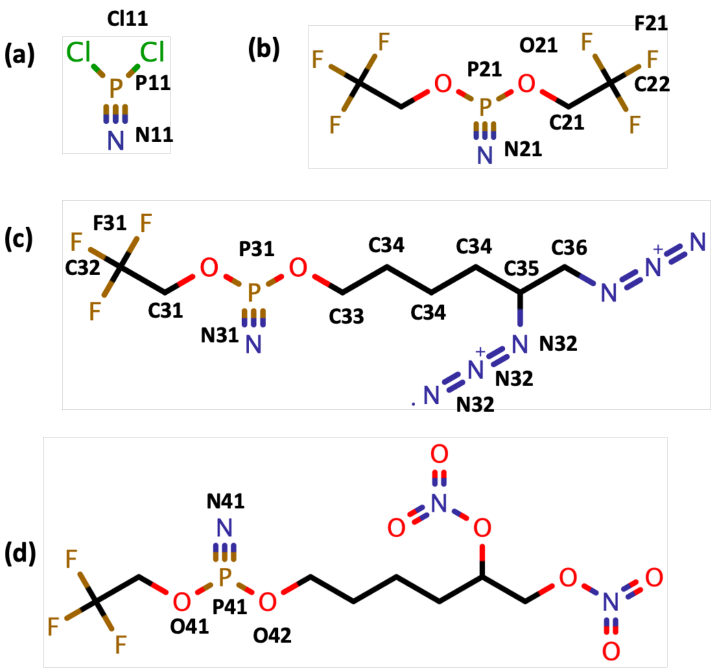

Table S1. List of the partial atomic charges for the unique atomic sites for the PZ-DC (the labels corresponding to Figure S1).

Label	q / esu
**P11**	0.1044
**N11**	0.0706
**Cl11**	-0.0875

Table S2. List of the partial atomic charges for the unique atomic sites for the PZ-TFE (the labels corresponding to Figure S1).

Label	q / esu	Label	q / usu
**P21**	0.0390	**C22**	0.1841
**N21**	0.0788	**F21**	-0.0981
**O21**	-0.2526	**H21**	0.1708
**C21**	-0.0377		

Table S3. List of the partial atomic charges for the unique atomic sites for the PZ-Azido (the labels corresponding to Figure S1).

Label	q / esu	Label	q / usu
**P31**	0.0493	**C36**	-0.1503
**N31**	0.0432	**N32**	-0.0550
**O31**	-0.2952	**F31**	-0.1176
**O32**	-0.3181	**H31**	0.1535
**C31**	-0.0270	**H33**	0.1487
**C32**	0.1478	**H34**	0.1416
**C33**	-0.0063	**H35**	0.1441
**C34**	-0.2158	**H36**	0.1455
**C35**	-0.0023		

Table S4. List of the partial atomic charges for the unique atomic sites for the PZ-Nitrato (the labels corresponding to Figure S1).

Label	q / esu	Label	q / usu
**P41**	0.0432	**N42**	0.8215
**N41**	0.0837	**O43**	-0.5148
**O41**	-0.2570	**O44**	-0.4042
**O42**	-0.3022	**F41**	-0.0994
**C41**	-0.0354	**H41**	0.1689
**C42**	0.1832	**H43**	0.1453
**C43**	-0.0186	**H44**	0.1582
**C44**	-0.2213	**H45**	0.1821
**C45**	0.1514	**H46**	0.1706
**C46**	0.0163		

Table S5. Bond length and bond force constants used for relaxing newly formed bonds between the monomers for each system.

Step	r_0 / Å	k_b / kcal.mol^{-1}	Step	r_0 / Å	k_b / kcal.mol^{-1}
**1**	6.00	1	**11**	3.50	140
**2**	5.75	2	**12**	3.25	160
**3**	5.50	5	**13**	3.00	180
**4**	5.25	10	**14**	2.75	200
**5**	5.00	20	**15**	2.50	225
**6**	4.75	40	**16**	2.25	250
**7**	4.50	60	**17**	2.00	275
**8**	4.25	80	**18**	1.75	300
**9**	4.00	100	**19**	1.495	325
**10**	3.75	120	**20**	1.495	350

Figure S2. (a) Tensile stress-strain curves (SSCs) for the polymerised systems obtained at 20 °C. (b) A zoomed-in version of (a). The lines represent the fit lines used to calculate the Young's modulus, up to a strain of 2 %.

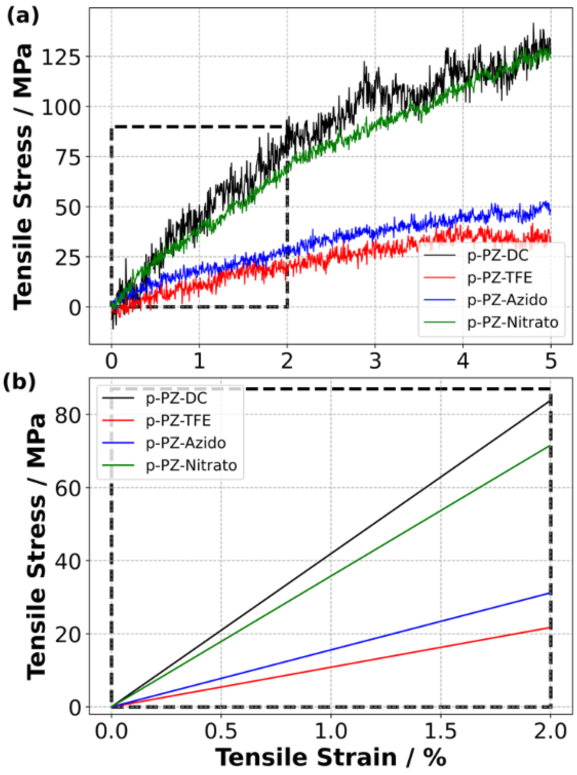

Supplement: Supplementary file 1 [file polymers-14-01451-s001.zip › polymers-1634122-supplementary.pdf]
